# Different Dimensions of the Home Food Environment May Be Associated with the Body Mass Index of Older Adults: A Cross-Sectional Survey Conducted in Beijing, China

**DOI:** 10.3390/nu16020289

**Published:** 2024-01-18

**Authors:** Man Zhang, Ruixin Chi, Zhenhui Li, Yujie Fang, Na Zhang, Qiaoqin Wan, Guansheng Ma

**Affiliations:** 1 School of Nursing, Peking University, 38 Xue Yuan Road, Haidian District, Beijing 100191, China; zhangman@bjmu.edu.cn; 2 Department of Nutrition and Food Hygiene, School of Public Health, Peking University, 38 Xue Yuan Road, Haidian District, Beijing 100191, China; crx@bjmu.edu.cn (R.C.);; 3 Laboratory of Toxicological Research and Risk Assessment for Food Safety, Peking University, 38 Xue Yuan Road, Haidian District, Beijing 100191, China

**Keywords:** home food environment, body mass index, BMI, older adults

## Abstract

Objective: The objective of this study was to evaluate the home food environment of the elderly in Beijing and analyze its association with the body mass index (BMI) of the elderly, as well as to provide recommendations for improving the home food environment for the elderly. Methods: This study was conducted in Beijing, China, in 2019. The participants were 1764 elderly individuals aged 65 to 80, recruited from 12 communities through a multistage stratified random sampling method. The study involved the use of questionnaire surveys to gather data on participants’ demographics, the availability of various foods in their households, and their living conditions. Socioeconomic status (SES) was evaluated based on their educational level, occupation, and income level. Height and weight measurements were taken to calculate BMI. We conducted both univariate analysis and multiple linear regression analysis to evaluate the relationship between the home food environment and BMI. Results: A total of 1800 questionnaires were distributed, of which 1775 were retrieved, resulting in a questionnaire recovery rate of 98.6%. Among these, 1764 questionnaires were deemed valid, corresponding to a questionnaire validity rate of 99.4%. The participants had a mean age of 69.7 ± 4.3 years old, over 40% of whom were overweight or obese. In terms of low-energy/high-nutrient-density foods, the most readily available items were fresh vegetables (95.6%), followed by coarse grains (94.1%), fresh fruits (90.4%), and dairy products (83.6%). Among high-energy/low-nutrient-density foods, preserved foods were the most available (51.9%), followed by salted snacks (40.6%), sugary beverages (28.2%), and fried foods (9.4%). Approximately 7.3% of participants lived alone. Elderly individuals with higher SES had a lower BMI compared to those with medium to low SES (25.9 vs. 26.5, 25.9 vs. 26.4, *p* < 0.05). Those living alone had a higher BMI than those who did not (27.2 vs. 26.2, *p* = 0.001). After controlling for potential confounding variables, older adults with high SES exhibited a BMI reduction of 0.356 kg/m^2^ (*p* = 0.001), whereas those living alone exhibited an increase in BMI of 1.155 kg/m^2^ (*p* < 0.001). The presence of preserved foods at home was linked to a BMI increase of 0.442 kg/m^2^ (*p* = 0.008). Conclusion: This study underscores the significant impact of family SES, living conditions, and the availability of preserved foods on the BMI of elderly individuals.

## 1. Introduction

The world is experiencing a rapid aging phenomenon compared to the past. China transitioned into an aging society in 2000 and currently hosts the largest population of older adults (aged 60 and above) worldwide [1]. The population of individuals aged 65 and above in China had reached 210 million by the end of 2022, accounting for 13.5% of the total population [2]. As the population ages, China has undergone a significant epidemiological shift from infectious diseases to non-communicable diseases (NCDs). Overweight and obesity are closely linked to increased morbidity from NCDs and cardiovascular disease mortality [3]. In recent decades, Chinese residents have undergone a dietary transition characterized by a move towards a diet rich in fats and energy but low in fiber [4]. Concurrently, the issues of overweight and obesity have come to the forefront. Based on the “Report on the Nutrition and Chronic Disease Status of Chinese Residents (2020)”, the rate of overweight individuals aged 60 and above increased from 31.9% in 2012 to 36.6% in 2018, and the obesity rate rose from 11.6% to 13.6% [5].

The occurrence and development of obesity are influenced by various factors, including genetic factors, dietary factors, environmental factors, etc. Among these factors, the food environment, as one of the significant influencing factors of obesity, has received increasing attention [6,7]. An ecological framework that illustrates the food environment encompasses individual-level factors, the social environment, the physical surroundings, and macro-level factors [8]. Given that people primarily store and consume food at home, numerous factors within the home food environment have been correlated with dietary behaviors and weight status. Globally, significant evidence has highlighted the associations between the home food environment and body mass index (BMI). However, there is currently no unified concept of the home food environment [9]. Richard R. divides the home food environment into three dimensions: political and economic environments, sociocultural environments, and built and natural environments [10]. As per the ANGELO framework, the obesogenic home environment comprises four dimensions: physical, economic, political, and sociocultural dimensions [11].

Researchers have extensively focused on the physical environment in the home, namely, the availability of food as the primary aspect of the home food environment [12]. While many of these investigations have centered on children, a few have concentrated on adults [12,13,14,15]. A study conducted among American adults revealed that a more diverse availability of fruits and vegetables was linked to lower odds of overweight/obesity [12]. Emery reported that homes of obese individuals had a lower availability of healthy food options in comparison to homes of non-obese individuals [13]. Gorin’s research similarly revealed that overweight adults had a reduced presence of low-fat snacks and fruits/vegetables but an increased presence of high-fat snacks compared to those with normal weight [14]. In the case of overweight and obese women, the quantity of unhealthy foods within the household was linked to the percentage of calories derived from fat [15]. Home food availability can serve as an effective target for interventions aimed at addressing overweight and obesity.

Socioeconomic status (SES) also has an influence on obesity. A meta-analysis primarily derived from studies conducted in developed countries revealed a correlation between SES and obesity in the female population [16]. In countries with low and middle incomes, individuals with higher SES are more likely to experience obesity [17]. Notably, none of the studies mentioned above were centered on the Chinese population. An investigation targeting Chinese children suggested a potential positive correlation between high SES and childhood obesity [18]. Furthermore, numerous other facets of the home food environment exhibit associations with obesity, including family size and structure, family members dining together, and household dining rules [8,19]. However, there is limited research focusing on these factors.

Currently, most studies in this area are centered on developed countries, with limited studies conducted in China. The distinctive social and cultural context in China may exert different effects on the correlation between the home food environment and the prevalence of overweight and obesity, highlighting the need for more China-specific research to bridge this knowledge gap. The influence of the home food environment may vary significantly based on the dietary habits of specific subgroups. Given the reduced mobility of older adults, their dietary habits and weight status might be more heavily influenced by their home food environment. While most studies have concentrated on children, research on older adults in this regard is notably scarce. Given the increasingly pressing aging demographic in China, prioritizing the health status of elderly individuals is of paramount importance. The incompleteness of current findings, along with the absence of Chinese-specific and older-adult-focused evidence, underscores the necessity for additional research.

In this study, we aimed to comprehensively characterize the home food environment across three dimensions and analyze its association with BMI in the elderly population. Ultimately, our goal was to provide insights and recommendations for the enhancement of the home food environment, thereby improving the health of the elderly.

## 2. Materials and Methods

### 2.1. Study Design

Our study employed a cross-sectional design, encompassing both urban and suburban areas of Beijing, China.

### 2.2. Participants

The participants were from a 2019 cross-sectional survey that focused on the dietary behaviors and factors influencing older adults. The recruitment of participants took place across 12 communities spanning 3 districts in Beijing. A total of 1800 questionnaires were distributed, with participant numbers ranging from 117 to 181 individuals per community. Participants included in the analysis met the criteria of providing complete information on age and gender, in addition to undergoing accurate height and weight measurements. Among these, 1775 were retrieved, resulting in a questionnaire recovery rate of 98.6%. In total, 1764 questionnaires were deemed valid, yielding a questionnaire efficiency rate of 99.4%.

Inclusion criteria for the study encompassed individuals who were between 65 and 80 years old, retired from employment, residing within a single community for at least two years, and functionally independent. Individuals unable to consume food normally and those with cognitive impairment were excluded from the study.

### 2.3. Sample

#### 2.3.1. Sampling Method

We utilized a multistage stratified random sampling approach. Initially, the survey targeted three districts: Haidian, Shunyi, and Miyun. These districts were selected to represent varying economic statuses and geographical locations within Beijing. Haidian District, positioned in close proximity to Beijing’s city center, exhibited the highest level of economic development, succeeded by Shunyi District and, finally, Miyun District. Subsequently, in each district, one urban street and one suburban street were chosen. Following this, two communities within each selected street were identified for study inclusion. Finally, a random selection of older adults residing in each of the chosen communities participated in the survey.

#### 2.3.2. Sample Size Calculation

For sample size calculation, we utilized the obesity rate among older adults in China from previous studies [20,21]. The sample size was determined using the formula N = Z_1−α/2_^2^ p(1 − p)/e^2^, where we set α = 0.05, Z_1−α/2_ = 1.96, and e = 0.03. The parameter p denotes the estimated obesity rate among the elderly population in China, which is 0.15.

Considering that we investigated three districts and accounting for a 10% anticipated dropout rate, the determined final sample size for validity was 1800 elderly participants.

### 2.4. Ethical Review

The study protocol underwent a thorough review and received approval from the Peking University Biomedical Ethics Committee (approval code: IRB00001052-17112). Adherence to the principles outlined in the Declaration of Helsinki was ensured throughout the study. Prior to their participation, all participants were furnished with an informed consent form, and their voluntary commitment to engage in the study was obtained through the act of signing the document. Subsequently, the researchers securely retained the written informed consent from each participant.

### 2.5. Measurements

#### 2.5.1. Participants’ Basic Information

Participant information was gathered via questionnaires, encompassing fundamental details including address; gender; age; educational level; marital status; income; living conditions; occupation; and habits related to exercise, smoking, and drinking. Please refer to Supplementary S1 for the specific content of the questionnaire. The questionnaire was completed individually, with the investigator and each participant filling it out in person.

The economic status of the community was determined based on the address participants filled out in the questionnaire and by querying the Beijing Statistical Zoning Code and Urban Rural Classification Code (2018 Edition) [22]. In this document, all communities in Beijing are coded. The code 111 represents urban areas, 112 represents suburban areas, and 200 represents rural areas. In this study, all communities originated from urban and suburban areas.

#### 2.5.2. Home Food Availability

The questionnaire employed to assess home food availability was derived from a previously published tool [23] and modified based on the results of expert consultation. In this study, a list of eight food items was provided and categorized into two groups based on their nutritional value and caloric density [19]. Among these, four are low-energy/high-nutrient-density foods, which are recommended for sufficient intake in the Dietary Guidelines for Chinese Residents, namely fresh fruits, fresh vegetables, dairy products, and coarse grains; while the other four are high-energy/low-nutrient-density foods, which should be consumed less or avoided, namely salted snacks, sugary beverages, preserved foods, and fried foods. See Table 1 for details of each food item.

To evaluate home food availability, participants were asked, “How frequently are the following food items available in your home?” Participants provided responses using a five-point scale, selecting from options including “always”, “most of the time”, “sometimes”, “occasionally”, or “never”. These response frequencies were further categorized into two groups: “high availability”, encompassing “always” and “most of the time”, and “low availability”, including “sometimes”, “occasionally”, and “never” [23].

#### 2.5.3. Food Intake Information

The participants’ food intake information was collected through a questionnaire. This questionnaire has been applied in many previous studies and proven effective [24,25,26]. In the questionnaire, the participants were asked to answer the following question: “Have you eaten this kind of food in the past three days?”. The food classification was based on the balanced diet pagoda of Chinese residents: cereals, vegetables, fruits, animal meat, fish and shrimp, eggs, milk and dairy products, beans and soy products, and oil and fat. Foods outside these 9 categories, such as carbonated beverages, alcoholic beverages, coffee, candy, etc., were not included in the survey. We only investigated whether the participants had eaten these foods and did not consider the frequency and amount of food intake. The answer options were classified as “yes” or “no”. For the 9 types of food we investigated, if the answer was “yes”, 1 point was assigned; if the answer was “no”, 0 points were assigned. The scores of the 9 food categories were summed to obtain the total DDS score. The minimum score was 0, and the maximum score was 9.

#### 2.5.4. Home Socioeconomic Status (SES)

In this study, we adopted the Programme for International Student Assessment (PISA, 2009) as a reference and integrated methods outlined in the published literature [27,28,29] to compute the SES of older individuals within their home environments. The specific steps are described as follows.

The initial step involved gathering data on the educational level, occupation, and income level of the elderly via a questionnaire survey. Subsequently, values were assigned to these data points. Educational level was graded in accordance with the criteria used by PISA, with, for instance, 6 points assigned to primary school and 9 points for junior high school. To determine occupation, we considered that the occupational classification within the International Socio Economic Status Occupational Classification Index (ISEI), as used by PISA, was not applicable to China. Consequently, we employed the occupational reputation index developed by Chinese scholar Li Chunling. This index encompasses a total of 161 occupations, each assigned a score ranging from 10.4 to 90 points [30]. Household per capita income in RMB was categorized into discrete points, with “below RMB 2000” earning 2 points, “RMB 2000~3499” earning 3.5 points, “RMB 3500~4999” earning 5 points, “5000~6499” earning 6.5 points, “6500~9999” earning 10 points, and “over RMB 10,000” earning 12 points.

The second step was to address missing values within the three variables. The following principle was used to handle missing data: if two or more variable values were missing within a sample, that sample was classified as missing data. In cases where only one variable value was missing, we utilized the available values of the remaining two variables to conduct regression estimation for the missing variable and subsequently replaced the missing value.

The third step involved calculating the SES score. After transforming the three mentioned variables into standardized scores, we utilized principal component analysis to calculate the SES through the application of the following formula: SES = (β1 × Z Educational level + β2 × Z Occupation + β3 × Z Income level)/εƒ. In this equation, β1, β2, and β3 represent factor loads, while εƒ denotes the characteristic root of the first factor. Participants’ total scores spanned from −2.89 to 2.98. Higher SES scores reflect a higher objective socioeconomic status of participants’ families.

In this study, home SES was categorized into three groups based on SES scores: low, medium, and high.

#### 2.5.5. Physical Measurement

The investigators received prior training. They conducted height and weight measurements using standardized procedures and uniform instruments (RGZ-160; Suheng, Jiangsu, China). Each participant was measured twice. Heights were recorded to the nearest 0.1 cm, and weights were recorded to the nearest 0.1 kg.

For height measurement [31], participants assumed a barefoot, erect posture on the altimeter’s floor, ensuring contact of their heel, sacrum, and shoulder blades with the altimeter’s base. With the head held upright and eyes looking straight ahead, the upper edge of the tragus and the lower edge of the orbit were aligned horizontally.

For weight measurement [31], participants wore as little clothing as possible and assumed a natural stance at the center of the scale. Data were read once participants achieved a stable stance.

### 2.6. Variables

#### 2.6.1. Body Mass Index

After determining the mean height and weight, the BMI was calculated and expressed in kg/m^2^ to describe the characteristics of the sample. According to The Dietary Guidelines for Chinese Residents (2022) [32], we defined a BMI below 20.0 as underweight, the range from 20.0 to 26.9 as normal weight, and a BMI above 27.0 as overweight or obese.

#### 2.6.2. Home Food Environment Variables

Living conditions were grouped into “living alone” and “not living alone”. Socioeconomic status (SES) was classified into three categories: “high”, “medium”, and “low”. Family food availability encompassed a total of eight food items, each classified as binary, indicating “high availability” or “low availability”.

#### 2.6.3. Confounders

Confounding variables in our study were identified through a comprehensive literature review and expert discussions. These included individual-level sociodemographic characteristics, such as age, gender, and marital status. Additional behavioral factors were also considered, such as exercise frequency, smoking and drinking habits, dietary diversity score (DDS), and the economic status of the communities.

### 2.7. Statistical Methods

SPSS Statistics 20.0 (IBM Corp., Armonk, NY, USA) was used for statistical analyses. Descriptive statistics were employed to examine participant and home food environment characteristics. Measurement data are reported as mean and standard deviation, whereas enumeration data are represented in terms of frequency and percentage.

We conducted a univariate analysis of BMI to compare the BMI of older adults with different characteristics. We also used multiple linear regression analysis to investigate the correlation between each home food environment variable and BMI. Model 1 included solely the home food environment variables, while Model 2 incorporated confounders including demographic characteristics, neighborhood socioeconomic level, and behavioral factors. A significance level of *p* < 0.05 was considered statistically significant.

## 3. Results

### 3.1. Participant Characteristics

This sample was from the same study as our previously published article. As described in our previous article, a total of 1800 questionnaires were distributed, of which 1775 were retrieved, resulting in a questionnaire recovery rate of 98.6%. Among these, 1764 questionnaires were deemed valid, corresponding to a questionnaire validity rate of 99.4%. Among the participants (*n* = 1764), the average age was 69.7 ± 4.32 years old, and the male-to-female ratio was approximately 1 to 1.4. The mean BMI was 26.3 ± 3.50 kg/m^2^, with over 40% of elderly individuals being overweight or obese. Participants were approximately evenly distributed between urban and suburban areas. Only 17% of the elderly population reported smoking habits, and 24.7% consumed alcohol once a week or more. For a more detailed overview of the participants’ basic information, please refer to Table 2 [33].

### 3.2. Home Food Availability

**Figure 1:**
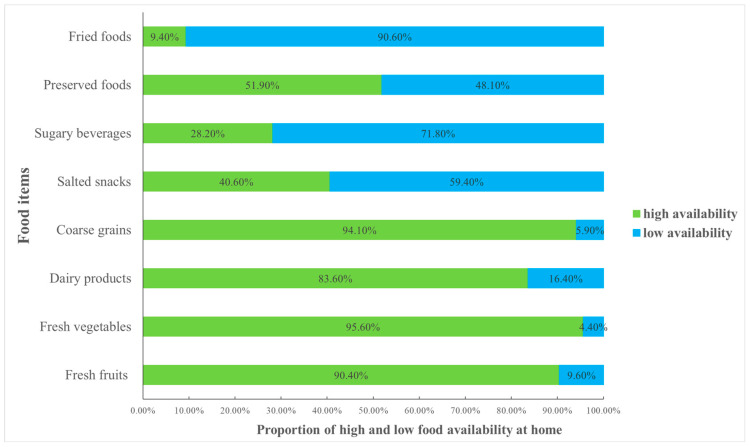
The proportion of high and low food availability of different food items in the home.

Among the four low-energy/high-nutrient-density foods, the highest to lowest availability was fresh vegetables (1686, 95.6%), coarse grains (1660, 94.1%), fresh fruits (1594, 90.4%), and dairy products (1475, 83.6%). Among the four high-energy/low-nutrient-density foods, the availability, ranked from highest to lowest, was preserved foods (915, 51.9%), salted snacks (717, 40.6%), sugary beverages (498, 28.2%), and fried foods (165, 9.4%) (see Figure 1).

### 3.3. Living Conditions

**Figure 2:**
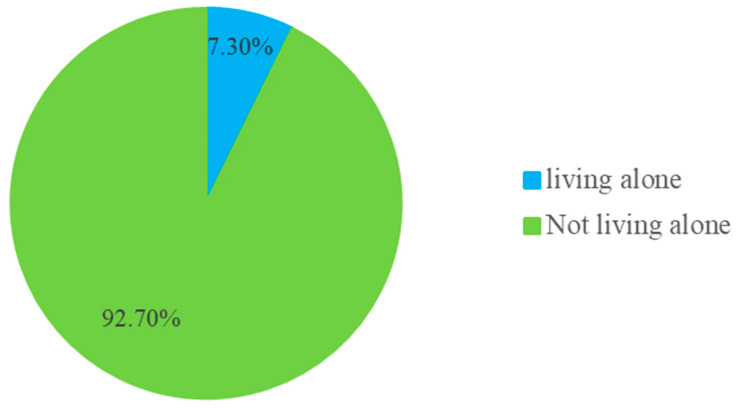
Proportion of older adults with different living conditions.

Among all participants, 7.3% lived alone (see Figure 2).

### 3.4. Univariate Analysis of BMI

The results of univariate analysis of BMI are presented in Table 3. The BMI of older adults varied with SES. Subsequent pairwise comparisons revealed that elderly individuals with high SES had lower BMI compared to those with medium to low SES (25.9 vs. 26.5, 25.9 vs. 26.4, *p* < 0.05). Older adults living alone exhibited higher BMI values compared to those not living alone (27.2 vs. 26.2, *p* = 0.001). No significant differences in BMI were observed among elderly individuals with either high or low availability of various foods in their homes (*p* > 0.05).

### 3.5. Association between Home Food Environment and BMI

After adjusting for potential confounding factors, our analysis revealed several noteworthy correlations. Specifically, we observed a negative correlation between family SES and BMI. Older adults with higher SES had a reduced BMI of 0.356 kg/m^2^ (*p* = 0.001) when compared to those with lower SES. Furthermore, living condition emerged as a key contributor, as older adults living alone experienced an elevation in BMI of 1.155 kg/m^2^ (*p* < 0.001) compared to their counterparts who did not live alone. Additionally, we found that the availability of preserved foods was positively associated with BMI, indicating that older adults with higher availability of preserved foods experienced an increase in BMI of 0.442 kg/m^2^ (*p* = 0.008) (see Table 4 for more details).

## 4. Discussion

The home serves as the paramount food environment, as the majority of food storage, preparation, and consumption occurs within its confines. Our findings indicate that diverse factors of the home food environment might be linked to BMI among elderly individuals.

According to our results, higher availability of preserved foods within the home was associated with a higher BMI among older adults. In many regions of China, preserved foods constitute an integral part of family diets, including items like preserved vegetables, preserved meat, and preserved fish. Existing research evidence has suggested that frequent consumption of preserved foods may be linked to various health issues, such as overweight, obesity, hypertension, fatty liver, primary liver cancer, and upper gastrointestinal cancer [34,35,36]. A study involving civil servants aged 30 to 50 also identified frequent consumption of preserved foods as a risk factor for overweight and obesity, while regular intake of fresh vegetables was found to be a protective factor against these conditions [37]. Preserved foods are typically characterized by their high salt content, and older adults may also consume more grains when partaking in such foods, potentially leading to excessive energy intake and an increase in BMI. Consequently, it is advisable to reduce the availability of preserved foods in the home, placing a stronger emphasis on consuming fresh vegetables, meat, and other fresh food items. However, it is worth noting that in this study, preserved foods were not further categorized into subtypes like preserved meat, preserved vegetables, or other preserved foods. Further research is warranted to determine which specific types of preserved foods have a significant impact on BMI.

In this study, a high SES within the household was found to be associated with lower BMI among the elderly. This finding aligns with previous research conducted in developed countries [16] but contrasts with results observed in low-income countries [17]. It is noteworthy that this investigation was conducted in Beijing, the capital of China, which is known for its elevated economic status. To establish a more comprehensive understanding, future research should encompass diverse regions within China. Interestingly, this outcome contradicts prior findings in Chinese children [18], highlighting the potential variability in the influence of SES on BMI across different population groups. This underscores the need for more targeted research specifically focusing on the elderly to better elucidate these dynamics.

Our study revealed that living alone is a contributing factor to increased BMI in older adults—a finding consistent with prior research. In a study involving older Japanese adults, the adjusted prevalence ratios for obesity (BMI ≥ 30.0 kg/m^2^) indicated that men who exclusively dined alone had a ratio of 1.34 (1.01–1.78) if they lived alone and 1.17 (0.84–1.64) if they lived with others [38]. Additionally, a retrospective cohort study conducted among university students identified living alone as a significant predictor of weight gain and overweight/obesity [39]. Moreover, research involving children and adolescents revealed that those participating in family meals at a frequency of at least three times per week had an increased likelihood of maintaining a normal weight, with a corresponding 12% decrease in the odds of overweight [40]. Individuals living alone face fewer opportunities to dine with others. While intervening in older adults’ living conditions can be challenging, encouraging communal eating rather than solitary dining is a feasible behavioral intervention. Community kitchen tables and food delivery services at the community level can serve as potential solutions. The Chinese government has made commendable efforts to date to establish such facilities and services. However, there remain shortcomings, including limited service variety, suboptimal service quality, constraints in terms of site scale, and suboptimal site layouts [41,42,43]. It should be noted that in this study, the sample size for individuals living alone was relatively small (7.30%), which may have introduced a certain degree of bias. Moreover, the small sample size may have affected the representativeness and generalizability of the results. Specifically, for the population living alone, this limitation in terms of sample size could have led to an overestimation or underestimation of the impact on BMI. Therefore, future research should include a larger number of individuals living alone to validate our findings and to more accurately investigate the relationship between living alone and BMI. Additionally, given the potential bias introduced by the small sample size, we should exercise caution in drawing conclusions.

Our study contributes valuable insights to the growing body of evidence regarding the possible impact of the home food environment among Chinese older adults. Although our study found that SES and the availability of preserved foods have only a slight impact on BMI, these findings are still meaningful. First, research has shown that even minor weight loss, particularly for individuals who are overweight or obese, can reduce the risk of chronic diseases such as cardiovascular disease and diabetes [44,45]. Second, the current results provide an indication of potential trends, laying a foundation for future health education and intervention measures. This research highlights specific target groups for future health education and interventions, notably individuals living alone and those with lower SES. Enhancing food availability within the homes of the elderly, with a particular emphasis on addressing the presence of preserved foods, should be a priority. Achievement of this goal can be facilitated through educational efforts targeting not only the elderly themselves but also their families and peers; the government can play a pivotal role in addressing this issue by implementing a range of measures. These measures may include improving community kitchen facilities and delivery services; reducing the prices of foods characterized by low energy and high nutrient density, such as fresh vegetables and fruits and dairy products; and providing food vouchers for older adults, among other strategies. The combined implementation of these measures can enhance the home food environment for the elderly, ultimately contributing to better health outcomes in this demographic. Additionally, this study offers directions for future research. Future studies should use standardized research tools and methods to examine the home food environment, delving deeper into the differences in food environments across different countries and their impact on BMI and associated health risks.

Our study boasts several notable strengths across various aspects, including innovation, variable measurements, sampling method, and statistical analysis [46]. (1) Insofar as we know, this is a pioneering exploration investigating the correlation between the home food environment and BMI among elderly individuals within the Chinese context. Our research not only contributes empirical evidence on this association but also lays a scientific foundation for policymakers in China. (2) Within the scope of this research, we comprehensively considered various dimensions of the home food environment, encompassing physical, economic, and social aspects. (3) In our study, we employed standardized procedures to measure participants’ height and weight directly, enhancing the accuracy of BMI calculations compared to relying on self-reported data. (4) We imposed no restrictions on participants with existing health conditions or obesity, mitigating the potential for selection bias in our sample. (5) Employing a multistage stratified random sampling method, our study effectively addressed neighborhood self-selection bias. (6) Our statistical model includes a wide array of potential confounding factors, encompassing demographic characteristics such as age, gender, and marital status, as well as neighborhood socioeconomic status and behavioral attributes like drinking, smoking, and exercise frequency. This comprehensive approach enhances the robustness of our findings.

Several limitations are inherent in our study. To begin with, the geographical scope was confined solely to the city of Beijing. Therefore, the findings may not be entirely representative of China as a whole. Despite implementing a multistage stratified random sampling method that encompassed diverse economic strata and geographic locations within Beijing, the study predominantly reflects the conditions specific to this city. Given that Beijing is characterized by a high level of economic development in China, our findings may not be applicable to the broader Chinese context. Thus, it is essential to replicate our findings in other regions, especially in cities with varying economic statuses. Secondly, due to the constrained number of questions in our questionnaire, we included only eight food items in our assessment. Four types of low-energy/high-nutrient-density foods are recommended for adequate intake in the Dietary Guidelines for Chinese Residents, while four types of high-energy/low-nutrient-density foods are advised to be consumed less or avoided. In this study, we used categories rather than individual foods. Although this made the food list less detailed, it also ensured that that important foods were not missed. Thirdly, in the complex home environment, we included indicators from only three dimensions: availability of eight food categories, living conditions, and socioeconomic status (SES). Future research should aim for a more comprehensive assessment by including additional relevant factors. Moreover, there is a need for the development of authoritative home food environment questionnaires tailored to different populations. Lastly, like many studies in this field, our research employed a cross-sectional approach. Therefore, it is important to emphasize that causal inferences should not be drawn from our findings.

## 5. Conclusions

Our study suggests that socioeconomic status (SES), living conditions, and the availability of preserved foods at home may potentially impact the BMI of older adults. However, the complexity of this relationship necessitates a cautious and detailed approach in any potential intervention measures or policy recommendations. Further research is advised to build upon these preliminary findings, guiding effective and contextually appropriate strategies to support the health of the elderly.

## Figures and Tables

**Table 1:** Food items used to asses home food availability.

Food Item	Concept and Examples
Fresh fruits	Natural, fresh, unprocessed fruits, such as apples, bananas, strawberries, watermelons, oranges, etc.
Fresh vegetables	Natural, fresh, unprocessed vegetables, such as leafy vegetables, fresh beans, rhizomes, melons, eggplant fruits, bacteria, algae, etc.
Dairy products	A variety of dairy products, such as fresh milk, cheese, yogurt, butter, cream, etc.
Coarse grains	Whole grains or cereals that have not been refined, including brown rice, quinoa, barley, oats, millet, buckwheat, etc.
Salted snacks	A category of foods that are typically savory in taste and contain added salt or salt-based flavorings, including potato chips, pretzels, popcorn, crackers, meat snacks like beef jerky, etc.
Sugary beverages	Drinks sweetened with various forms of sugar or sweeteners, including sodas, fruit juices, energy drinks, sweetened teas, flavored coffees, etc.
Preserved foods	Foods typically made by preservation with sugar or salt, including pickled vegetables, salted fish, salted eggs, salted meat, etc.
Fried foods	Various foods that are cooked by being submerged in hot oil or fat, such as French fries, fried chicken, tempura, onion rings, etc.

**Table 2:** Participant characteristics (*n*, %) [33].

Item	Classification	*n*	%
BMI (kg/m^2^)	Underweight: below 20.0	45	2.6
	Normal: 20–26.9	950	53.8
	Overweight and obese: 27.0 and above	769	43.6
Gender	Male	730	41.4
	Female	1034	58.6
Age	65–69	983	55.7
	70–74	483	27.4
	75–79	298	16.9
Marital status	Unmarried	3	0.2
	Married	1482	84.0
	Widowed	260	14.7
	Separated or divorced	19	1.1
Educational level	Higher education	190	10.8
	Secondary education ^1^	923	52.3
	≤Primary education	651	36.9
Income level (RMB) ^2^	≤2000	409	23.2
	2000–3500	652	37.0
	3500–5000	376	21.3
	5000–10,000	223	12.6
	≥10,000	24	1.4
	Missing	80	4.5
Neighborhood socioeconomic level	Urban	899	51.0
	Suburban	865	49.0
Frequency of exercise	Never	198	11.2
	1–2 times per week	55	3.1
	3–4 times per week	83	4.7
	5–6 times per week	35	2.0
	Every day	1387	78.6
	Missing	6	0.3
Smoking	No ^3^	1464	83.0
	Yes	300	17.0
Drinking	Once a week or more	436	24.7
	Less than once a week	1325	75.1
	missing	3	0.2

^1^ Including junior high school, senior high school, and various specialized secondary schools. ^2^ Per capita monthly income of households in RMB. ^3^ “No” indicates never smoking or having quit smoking.

**Table 3:** Univariate analysis of BMI.

Variables	Group	*n*	BMI	*t*/*F*	*p*
SES	High	588	25.9 ± 3.4	5.403	0.005
	Medium	588	26.5 ± 3.5		
	Low	588	26.4 ± 3.6		
Living condition	Living alone	128	27.2 ± 3.8	−3.237	0.001
	Not living alone	1636	26.2 ± 3.5		
Availability of fresh fruits	High	1594	26.3 ± 3.5	−1.738	0.082
	Low	170	25.8 ± 3.6		
Availability of fresh vegetables	High	1686	26.2 ± 3.5	1.392	0.164
	Low	78	26.8 ± 3.7		
Availability of dairy products	High	1475	26.2 ± 3.5	1.019	0.308
	Low	289	26.5 ± 3.4		
Availability of coarse grains	High	1660	26.3 ± 3.5	−0.960	0.337
	Low	104	26.0 ± 3.3		
Availability of salted snacks	High	717	26.2 ± 3.5	1.080	0.280
	Low	1047	26.4 ± 3.5		
Availability of sugary beverages	High	498	26.2 ± 3.5	0.446	0.656
	Low	1266	26.3 ± 3.5		
Availability of preserved foods	High	915	26.4 ± 3.4	−1.772	0.076
	Low	849	26.1 ± 3.6		
Availability of fried foods	High	165	26.5 ± 3.7	−0.783	0.433
	Low	1599	26.2 ± 3.5		

**Table 4:** Association between home food environment and BMI.

Variable	Model 1 ^1^			Model 2 ^2^		
	β	SE	*p*	β	SE	*p*
SES	−0.280	0.104	0.007	−0.356	0.108	0.001
Living condition	1.053	0.321	0.001	1.155	0.319	<0.001
Fresh fruits	0.865	0.297	0.004	-		
Fresh vegetables	−0.902	0.417	0.031	-		
Dairy products	-			-		
Coarse grains	-			-		
Salted snacks	-			-		
Sugary beverages	-			-		
Preserved foods	-			0.442	0.166	0.008
Fried foods	-			-		

Note. SE: standard error. ^1^ Model 1 represents the unadjusted model, including solely the home food environment variables. ^2^ Model 2 is adjusted for age, exercise frequency, smoking habits, and neighborhood socioeconomic level. Covariates are not displayed in the table for conciseness.

## Data Availability

The data presented in this study are available on request from the corresponding author. The data are not publicly available as the authors are currently engaged in further exploration and analysis of these data, and hence have chosen not to disclose them publicly at this stage.

## Supplementary Materials

The following supporting information can be downloaded at: https://www.mdpi.com/article/10.3390/nu16020289/s1, Supplementary S1: Questionnaire on basic information and Home food availability.
