# Supplementary material for: Different Dimensions of the Home Food Environment May Be Associated with the Body Mass Index of Older Adults: A Cross-Sectional Survey Conducted in Beijing, China
Source: Nutrients. 2024 Jan 18;16(2):289. doi: 10.3390/nu16020289 (PMC10821192; doi:10.3390/nu16020289)
Supplement: Supplementary file 1 [file nutrients-16-00289-s001.zip › nutrients-2686859-supplementary.pdf]

Supplementary S1

Questionnaire on basic information and Home food availability

Hello, dear residents!

Please answer the following questions independently and truthfully. There is no right or wrong answer, each of your answers is very important to us. At the same time, we will keep you confidential, please fill in truthfully according to your own real thoughts. Thank you for your cooperation!

Basic Information

1. Your address: _____District _____Street _____Community
2. Your gender: ①Male ②Female
3. Your age: _____ years old
4. Height: _____ meters (Must be filled out by the investigator after measurement)
5. Weight: _____ kg (Must be filled out by the investigator after measurement)
6. Your highest education level:
   ① Master's degree and above ② University or college
   ③ Secondary technical school, vocational school ④ High school
   ⑤ Junior high school ⑥ Elementary school
   ⑦ Did not graduate from elementary school ⑧ Did not go to school
7. Your marital status:
   ① unmarried ② married ③ widowed ④ divorced or separated
8. What is the monthly income per capita in RMB (after tax) of your family?
   ① Below 2000 ② 2000-3500 ③ 3500-5000
   ④ 5000-6500 ⑤ 6500-10000 ⑥ 10,000 and above
9. Who have you been living with in the past year? (Multiple choices are allowed)
   ① Living alone ② Living with spouse ③ Living with children
   ④ Other (please specify: __________)
10. What was your longest career before retirement
    ① Senior professional and technical workers (doctors, professors, lawyers, architects, engineers, etc.)
    ② General professional and technical workers (midwives, nurses, teachers, editors, photographers, etc.)
    ③ Managers/administrative officials/managers (factory directors, government officials, directors, administrative officials, etc.)
    ④ General office staff (secretaries, clerks)
    ⑤ Production personnel in agriculture, forestry, animal husbandry, fishing, and water conservancy industries
    ⑥ Workers (section leaders, team leaders, craftsmen, ordinary workers, lumberjacks, etc.)
    ⑦ Officer
    ⑧ Soldiers and Police

⑨ Service industry personnel (drivers, chefs, waiters, gatekeepers, barbers, salespeople, etc.)

⑩ Athletes, actors, performers

⑪ Other (please specify: __________)

11. In the past month, how often did you exercise? (Walking, running, dancing, ball games, etc.)

    ① Never ② 1-2 times a week ③ 3-4 times a week ④ 5-6 times a week ⑤ every day

12. Do you smoke?

    ① Never smoked ② keep smoking, never tried to quit smoking

    ③ have failed to quit smoking ④ have quit smoking

13. How often did you drink alcohol in the past year?

    ① Drink almost every day ② 3-4 times a week ③ 1-2 times a week

    ④ 1-3 times a month ⑤ Less than once a month ⑥ Never

Food intake information

14. Have you eaten this kind of food in the past three days?

Cereals	①Yes ②No
Vegetables	①Yes ②No
Fruits	①Yes ②No
Animal meat	①Yes ②No
Fish and shrimp	①Yes ②No
Eggs	①Yes ②No
Milk and dairy products	①Yes ②No
Beans and soy products	①Yes ②No
Oil and fat	①Yes ②No

Home food availability

15. How frequently are the following food items available in your home?

Fresh fruits	①always ②most of the time ③sometimes ④occasionally ⑤never
Fresh vegetables	①always ②most of the time ③sometimes ④occasionally ⑤never
Dairy products	①always ②most of the time ③sometimes ④occasionally ⑤never
Coarse grains	①always ②most of the time ③sometimes ④occasionally ⑤never
Salted snacks	①always ②most of the time ③sometimes ④occasionally ⑤never
Sugary beverages	①always ②most of the time ③sometimes ④occasionally ⑤never
Preserved food	①always ②most of the time ③sometimes ④occasionally ⑤never
Fried foods	①always ②most of the time ③sometimes ④occasionally ⑤never
